# An Affordable Insole-Sensor-Based Trans-Femoral Prosthesis for Normal Gait

**DOI:** 10.3390/s18030706

**Published:** 2018-02-27

**Authors:** Srinivas Pandit, Anoop Kant Godiyal, Amit Kumar Vimal, Upinderpal Singh, Deepak Joshi, Dinesh Kalyanasundaram

**Affiliations:** 1 Centre for Biomedical Engineering, Indian Institute of Technology Delhi, New Delhi 110016, India; ird11831@cbme.iitd.ac.in (S.P.); bmz138038@cbme.iitd.ac.in (A.K.G.); bmz128466@cbme.iitd.ac.in (A.K.V.); dineshk.iitdelhi@gmail.com (D.K.); 2 Department of Physical Medicine and Rehabilitation, All India Institute of Medical Sciences, New Delhi 110029, India; usingh@aiims.ac.in; 3 Department of Biomedical Engineering, All India Institute of Medical Sciences, New Delhi 110029, India

**Keywords:** lower limb prosthesis, trans-femoral amputee, MR damper, knee damping control

## Abstract

This paper proposes a novel and an affordable lower limb prosthesis to enable normal gait kinematics for trans-femoral amputees. The paper details the design of a passive prosthesis with magneto-rheological (MR) damping system and electronic control. A new control approach based on plantar insole feedback was employed here. Strategically placed sensors on the plantar insole provide required information about gait cycle to a finite state controller for suitable action. A proportional integral (PI) based current controller controls the required current for necessary damping during gait. The prosthesis was designed and developed locally in India keeping in view the cost, functionality, socio-economic, and aesthetic requirements. The prototype was experimentally tested on a trans-femoral amputee and the results are presented in this work. The implementation of the proposed design and control scheme in the prototype successfully realizes the notion that normal gait kinematics can be achieved at a low cost comparable to passive prostheses. The incurring cost and power expenditure of the proposed prosthesis are evaluated against passive and active prostheses, respectively. The commercial implications for the prosthesis were explored on the basis of recommendations of ISPO Consensus Conference on Appropriate Prosthetic Technology in Developing Countries. The key objective of this work is to enable lucid design for development of an affordable prosthesis in a low-resource setting.

## 1. Introduction

About 30 million amputees are residing in low income countries [1,2]. Among those, only 5% to 15% are capable to receive a much-needed prosthesis [3]. Although the popular state-of-the art prostheses used in developed countries offer several advanced features, its application is hindered in low income developing countries primarily due to high cost, different functional demands, cultural issues, and unavailability of components locally [4,5]. 

Gait parameters of trans-femoral amputees including step length, inter-leg symmetry, hip exertion, and involvement of upper body are highly affected [6]. Over the years, there have been considerable efforts to develop affordable prostheses for trans-femoral amputees in developing countries [6,7,8,9]. These efforts have resulted in a variety of lower limb prosthetic designs at reduced cost. However, these prosthetic designs include low-end technologies like manual locking knee joints, the weight activated braking mechanism, and polycentric knee joint mechanisms [2,6,10,11,12]. In manual-locking-knee joints, knee stability is improved by providing more stiffness which renders lower speed and increased energy expenditure during gait [10]. Problems associated with stiff-legged gait were overcome by incorporating weight activated brakes with a knee joint. A typical weight activated arrangement suffers from limitations of abnormal gait and delayed initiation of swing phase [10]. Polycentric knee joint mechanisms were quite useful in developed countries and now, with some modifications, are being developed to suit the needs of low-income countries. The polycentric knee joint linkages can be configured to improve the stability in the stance phase with fair swing phase initiation. It does impose a limitation. As the extent of stability is increased in the stance phase, the swing phase initiation gets delayed proportionally [8,13]. In this regard, some recent prostheses such as Le Torneau polycentric knee (Limbs International, El Paso, TX, USA), Stanford-Jaipur leg (Stanford University, Stanford, CA, USA), Remotion Knee (D-Rev), SASPL knee joint LCKnee (Andrysek et al.) and a three axes knee (Arelekatti et al.) at MIT [2,10,11,12] have been developed.

Le Torneau knee and Stanford-Jaipur knee joints have a similar polycentric design of four-bar linkage with little to offer other than stance phase stability. On the other hand, Remotion Knee is the advanced version of Stanford-Jaipur knee with curvature mimicking to address late stance phase initiation [2,10]. Studies have revealed that such prosthetic designs though provide a low-cost solution to a grieving population, it does not take into account the need for an early stance flexion-extension and properly timed late stance flexion [11,12]. The basic biomechanical aspects, when overlooked, can result in many psychological, physiological, and socio-economic problems [14]. Abnormal and sub-normative gait puts immediate and extended costs in terms of psychological stigma and socio-economic distress, whereas in the longer term it results in musculoskeletal impairments and adversely affects the physiological equilibrium [15,16,17,18]. It suggests that the excessive focus on cost reduction alone cannot serve the desired purpose and low-cost prostheses must also have the ability to provide a gait kinematics closer to that of a healthy person. 

Also, Andrysek et al. and Arelekatti et al. addressed the problem of abnormal gait in low-cost passive prostheses. Andrysek et al. SASPL engages or disengages depending on the loading of the prosthetic limb during weight bearing but is not able to provide early stance flexion-extension and optimal swing phase damping [11]. Arelekatti et al. incorporated able-bodied gait kinematics using an automatic early stance lock for stability, a linear spring for early stance flexion-extension and swing control. The design suffers from few limitations such as the necessity of full knee extension at the end of swing phase and failure to lock the knee before stance phase that can lead to instability and risk of fall. Further, due to the lack of any clinical gait analysis, the study does not establish the extent of improvement in amputee kinematics towards normative gait [12]. It offers sufficient motivation to look for a new prosthesis design and control scheme that can enable able-bodied gait kinematics in trans-femoral amputees at an affordable cost in developing countries. So far, all developed prostheses designed for developing countries are primarily passive in nature, the authors believe that a variable damping can provide the subject with comfortable walking, thereby yielding the desired results at a low cost.

In this paper, a novel low cost lower limb prosthesis was developed and its clinical testing and analysis are presented. A new foot plantar insole feedback-based control strategy was developed and implemented to provide necessary damping using an MR damper. The prosthesis enables the amputee to realize the able-bodied normal gait kinematics and meets the functional, socio-economic, and aesthetic needs of a trans-femoral amputee and is affordable even in low-income economies. The paper also focuses on essential design features and requirements of a prosthesis to assess its commercial viability in these countries such as low cost, light-weight, functionality, biomechanically appropriate, durable, using locally available materials etc., based on the recommendations of ISPO Consensus Conference on Appropriate Prosthetic Technology in Developing Countries and elsewhere [19,20,21,22]. The details are discussed later in Section 5.7. The key objective of this work is to enable lucid design for development of an affordable prosthesis in low-income economies.

## 2. Prosthesis Design

This rationale of the prosthesis design is presented in this section. The trans-femoral prosthesis shall serve the major function of providing stability during stance phase and controlled flex-extension during the swing phase of a gait cycle. In the stance phase, the stability was ensured by providing suitable resistance to knee joint flexion while the user transfers body weight from the sound/healthy limb to the prosthetic limb. Similarly, the swing phase flex-extension was controlled by regulating walking speed [22]. Both the functions were achieved by including springs and dampers in the design [3]. Either a variable damping system or a system with multiple dampers is required for a variable damping system incorporated in state of art active prostheses, whereas multiple dampers are used in recent advanced passive prostheses. The multiple-damper approach inherently puts the design at disadvantage in terms of serviceability [10,11].

A study conducted by Herr and Wilkenfeld [23] serves as the theoretical background for our design to realize able-bodied gait kinematics. The authors used an MR knee prosthesis to adapt knee damping using local sensing of knee force, torque, and position. A few recent studies conducted by Xu et al. [24], Park et al. [25], and Fu et al. [26] have also attempted to design a lower limb prosthesis based on an MR damper. Complex sensing, data-driven AI control and lack of local climatic adaptations as well as increased cost of the prosthesis arising from various factors ranging from non-inclusion of off-the-shelf components or locally manufactured components increased the inherent cost, making it unsuitable for applications in low-income economies. Further, the control architecture based on stiff tracking of the knee angle should be avoided in order to enable the amputee to use the prosthesis more interactively, rather than reacting to it [24]. Therefore, the potential of prostheses based on MR damper remain untapped in such low-income economies due to the unavailability, complexity, and incurring cost of design and manufacturing [20,23,27]. 

It is proposed that a single damper with a controllable damping capacity, such as an MR damper, if used with suitably designed sensing modality, driving circuitry, and control architecture, can provide able-bodied gait kinematics yet at a cost below par with advanced passive prostheses. Here, strategically placed sensors on the plantar insole provide sufficient information for normal gait kinematics, thereby enabling the control of the current in the MR damper during the different phases of a gait cycle. This forms the basis for the design of a simple MR damper-based knee joint mechanism and its control with plantar insole feedback.

### 2.1. Mechanical Design

**Figure 1:**
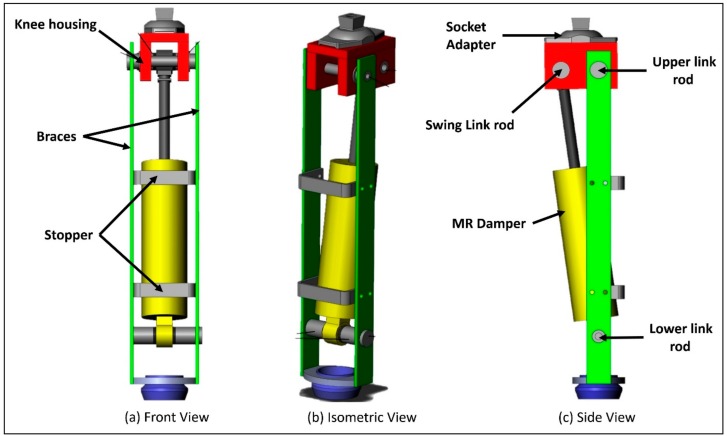
Three-dimensional structure and mechanical design of the proposed prosthesis.

The design of the prosthesis’ mechanical structure is shown in Figure 1. The design includes an MR damper, a hinge-type knee joint, and braces to support the leg assembly. In the MR damper, modulation of magnetic field strength can offer variable resistance to the flow of MR fluid. Placement of electromagnets around the piston rod and MR fluid as a working medium inside a hydraulic cylinder can result in a variable damping [20,23,27]. Damping can be modulated by changing the current flowing in the coils of the electromagnet. A variety of MR dampers are available to suit lower limb prostheses. In this study, a commercially available MR damper (Make: Lord Corporation, Cary, NC, USA; Model RD-8041 with a stroke length of 74 mm, and extended length of 248 mm) was employed in the prototype.

The piston rod and outer cylinder of the MR damper were fixed to the knee joint and the shank individually with swing link and lower-link rods. The upper-link and lower-link rods are attached to the braces. The upper link rod and swing-link rod form the knee axis and the control axis of the prosthesis, respectively; the upper link rod along with knee body articulates the knee braces via the knee axis. The control axis primarily responds to the ground reaction forces that cause relative motion between the knee and the braces. The swing-link rod transfers the forces generated by the MR damper during locomotion. The biomechanical mechanism of the control axis has been extensively reported elsewhere [26]. The control of the prosthesis in both the stance and swing phases was achieved by the same mechanical assembly and no separate mechanical component was used to lock the knee. The upper part of the knee joint (knee body) was connected to a socket connector to fasten the socket properly. The braces along with MR damper form the shank of the leg. It was connected to a Sach foot with a shank connector. The socket and shank connectors are connected to the knee and ankle adapters used by prosthetists for proper alignment of the prosthesis to the individual amputee. Knee Movement has been restricted to 90 degrees with protective bars (stoppers) placed in the forward direction of the damper. Custom designed components were used for link rods, plates, braces, and knee joint to meet the design specifications. Proper motion of the knee joint was ensured by simulating the solid model design in Solid Works^®^. During the simulation along the entire knee range, there was no interference between the joint links, bars, plates, and braces. With proper machining of all the custom components, the mechanism was assembled by bolting the braces to the knee joint. The MR damper was shoulder-bolted with the links of the assembly. Bearings were employed for smooth motion of joint between knee body plates and link rods. Off-the-shelf components such as connectors, adaptors, and the Sach foot were employed to extend the design with existing prosthetic components.

### 2.2. Sensing Mechanism

**Figure 2:**
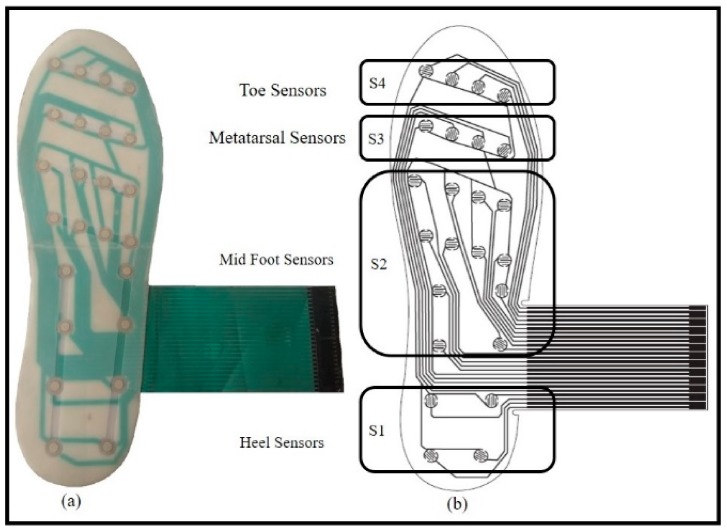
(**a**) Photograph of plantar insole and (**b**) sensor grouping.

**Figure 3:**
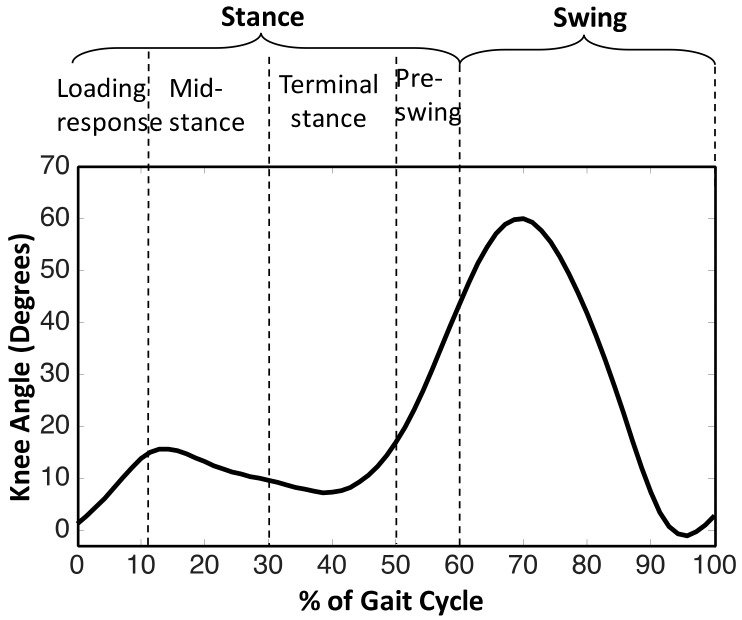
Typical gait cycle of a healthy person [33] and its classification [30].

Plantar insoles are extensively used for gait analysis of many locomotion impairments [28,29]. Although plantar insoles provide information during only the stance phase, it is sufficient for most of the rehabilitation support systems, posture and balance control [28]. Most of the plantar insoles employ a discrete number of sensors to obtain the required information; the minimum number of sensors vary from 2 to 16, or more depending upon the need [28]. Taking into account the advantages of foot plantar insoles, a novel sensing method was developed to control the prosthesis both in the stance and swing phases. It is composed of 24 switches strategically placed on the heel (S1), mid-foot (S2), metatarsal (S3), and toe (S4) of the plantar insole as shown in Figure 2. The sensor placement was determined by considering dominant pressure points of the plantar surface during walking as reported by Razak et al. [28]. The rationale for considering sensors on plantar insole lies in the wheel-like-behavior of plantar foot and its subsequently resulting roll-over shapes (ROS) [30,31]. Such ROSs are formed for various biomechanical events corresponding to different phases of a gait cycle of human locomotion i.e., loading response, mid-stance, terminal stance, pre-swing, and swing [32]. The progression of a typical gait cycle of a healthy individual along with subsequent phases and events are depicted in Figure 3. As the five segments are sequential in nature, the phases are distinctly noticed due to the formation of ROSs based on the combination of states of four groups or sensors placed on the foot plantar insole. The grouping of the 24 sensors into 4 groups is shown in Figure 2b. The stance phase events can be related to different combinations of the states of four sensor groups as tabulated in Table 1. If the state of any of the sensors in a group is high, then the state of the same group is assigned the value of 1.

The circuitry and mechanical dimensions of the insole were designed using Coral Draw^®^. The insole was printed using the silk screen printing process (Keywell Industrial Co., Noida, UP, India). In this process, silver-conducting ink and dielectric was used to print the circuit. While designing the insole, it was important to determine the minimum thickness of the dielectric layer and the amount of pressure that the insole may undergo during walking. It enables the designer to use an optimum thickness of the dielectric layer that is sufficient to trigger the events. For the initial prototype, this trade-off between minimum dielectric layer thickness and maximum loading was made using the ground reaction force (GRF) profile of the amputee on a force plate.

### 2.3. Embedded System

To feed the desired current to the MR damper at the given time instant, an electronic circuit that uses sensor readings was designed to regulate the current. The MR damper requires variable current to provide variable damping; the MR damper current can be varied by varying the input DC voltage across the load. It was achieved by a step-down DC/DC converter, controlled by a pulse width modulation (PWM) scheme. The duty cycle of the PWM signal was varied to reduce the error in the reference and actual feedback current. Error in reference and feedback current can be reduced to a permissible extent in desirable time by employing a proportional integral (PI) controller. The actual feedback current was measured with a Hall current sensor ACS712. The power circuit includes a battery bank rated at 9 V and 2.4 Ah, a power MOSFET, freewheeling diode, low pass LC filter, and MR damper. 

**Figure 4:**
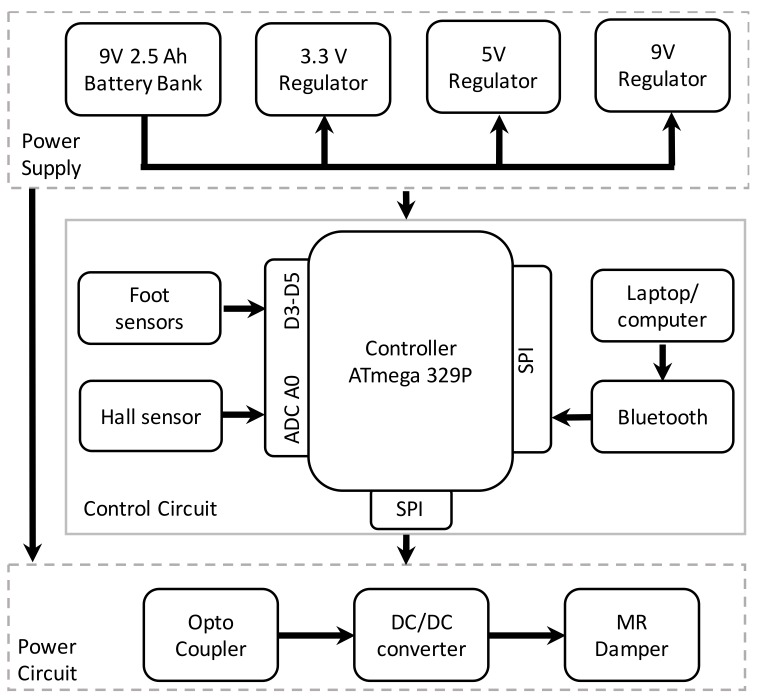
Embedded system layout.

The power and control circuits were isolated with an optocoupler and gate-driving circuit to ensure smooth operation. The control circuit includes the sensing mechanism along with a control unit as shown in Figure 4. A microcontroller is the central component of the control unit, that in-turn is the brain of the total signal processing system. The primary objective of the microcontroller is to control the power driving unit when required to change the damping. After careful analysis of the requirements, the Atmel mega (ATmega) 329P microcontroller was chosen for this work for its low cost, versatile characteristics, and more advanced features. The necessary processing of sensor signals and implementation of control algorithm was achieved with the firmware. The details of the components of the embedded system are given in Table 2.

### 2.4. Control Architecture

**Figure 5:**
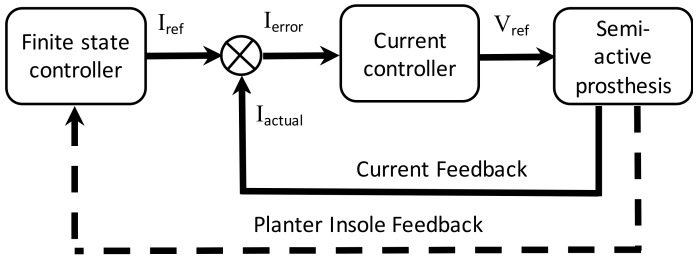
Block diagram of the control scheme.

A two-level control scheme was employed to achieve able-bodied gait kinematics as depicted in Figure 5. The control approach involves a finite state controller as a secondary controller and a conventional PI controller as a primary controller. The secondary controller generates the required current references for the MR damper using a finite state machine that ultimately modulates the impedance of the gait, depending on the gait event segment. The primary controller is a closed loop PI controller for MR damper current, which compensates for the load transfer dynamics, thus enabling the faithful tracking of current references with a higher bandwidth and accuracy as compared to its open loop counterparts. Prosthetic control is different from robotic control where the oscillatory response was avoided and the system was tasked to follow a stiff trajectory. PD, P, PID, sliding mode, and hysteresis controllers are inherently suitable for stiff tracking, whereas a PI controller offers a more suitable response. Furthermore, a PI controller is the most widely used current controller in industrial applications, and its incorporation would attract more commercial acceptability.

**Figure 6:**
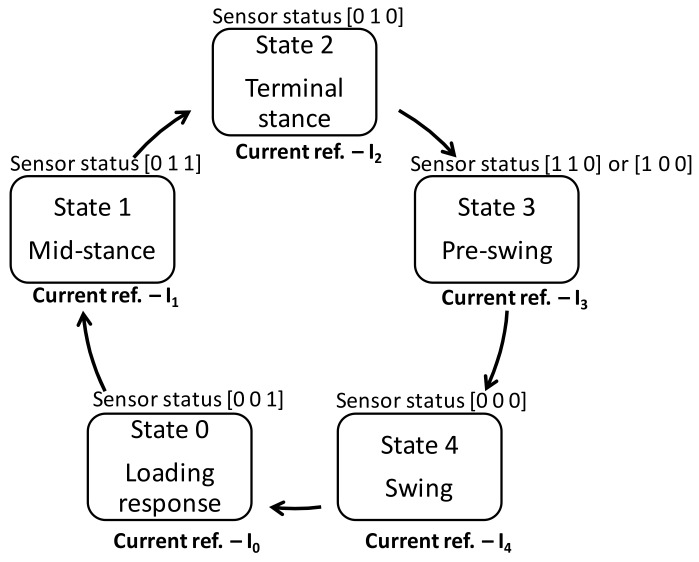
Finite state machine diagram.

This ordered behavior of state machines are utilized in neuro-prostheses with event triggered control [34,35]. The states of the finite state machine were determined by considering various demanded requirements [36]; here we intend to achieve the flexion-extension of the knee during the stance phase with a high degree of stability and a suitably damped swing phase to adjust the walking speed. In order to meet the stated requirements, a gait cycle with five segments/phases as described earlier were considered i.e., loading response, mid-stance, terminal stance, pre-swing, and swing. The above states are illustrated in Figure 6. Though there could be any number of states that can be used to form a state machine, as previous studies have reported, the introduction of a foot plantar insole with four sensor groups provides a total of 16 states, out of which 15 belong to stance phase and one to swing phase. Due to the ROS trajectory during level walking, only 7 states of stance can be assigned to four phases as tabulated in Table 1, whereas the remaining 8 states of stance do not represent any of the ROS segments. The available literature suggests that consideration of 4 states have successfully resulted in desired kinematics in the stance phase [27], whereas treatment of swing phase as a single state does not deviate much from the same [37].

In state 0, the knee flexes near to the maximum stance flexion. A relatively high damping was applied during this state to prevent buckling at the knee due to the user’s weight. During state 1, the knee begins to extend after maximum flexion. The rate of extension was lower than that of flexion during state 0, thereby requiring a high damping. The level of damping shall account for slow extension in order to avoid potential slamming of rotating parts to the knee housing and stoppers. State 2 involves the flexion-extension while the heel is off the ground and the sound limb is sharing the body weight, a moderate damping shall serve the purpose here. State 3 encounters the extension with a high rate of change and low body weight bearing; a low damping will provide the desired profile. The final state (state 4) represents a large knee flexion and associated extension after flexing to its maximum. The rate of change of flexion-extension suggests a very small damping for the same. All states can be tuned to suitable current references which can be accurately followed by a well-designed PI controller. The response of the PI controller must be fast and accurate as it lies in the inner loop of the control architecture.

## 3. Methods

The developed prosthesis was tested on a 24-year-old male (1.63 m, 60 kg) unilateral trans-femoral amputee in India as part of the target population. The subject was using a commercially available passive prosthesis (single axis extension assisting prosthetic knee joint) for the last three years post traumatic amputation. The amputee mobility predictor (AMP) score of the amputee was more than 35, i.e., above the K3 activity level. Table 3 shows the measurement of amputee and component used in the proposed prosthesis. A custom made Ischial containment socket was designed for the amputee by a licensed professional prosthetist. The prosthesis was assembled according to the measurements of the amputee. 

The dynamic alignment of the prosthesis was performed by the trained prosthetist who is one of the co-authors of the current work. The participant did not have any musculoskeletal injuries, sensory or neurological impairment or related disabilities other than trans-femoral amputation. The study was approved by the All India Institute of Medical Sciences ethics committee (Ref. No. IEC-35/09.02.17). The participant was explained the experiment protocols before starting the experiment and written consent was obtained.

### 3.1. Parameter Tuning and Training

**Figure 7:**
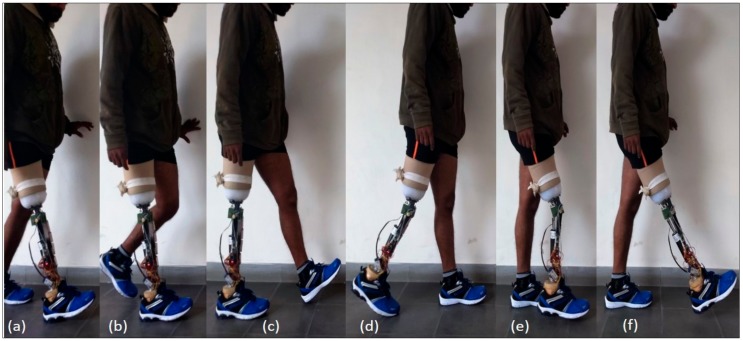
Photograph of subject with developed prosthesis during training in segment (**a**) loading response, (**b**) mid-stance, (**c**) terminal-stance, (**d**) pre-swing, (**e**) extended knee in swing, and (**f**) fully flexed knee in swing.

**Figure 8:**
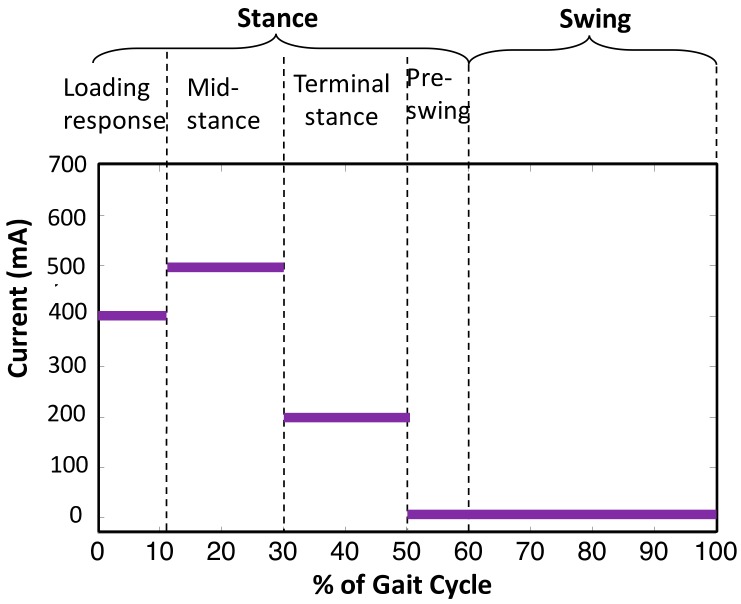
Final set of current references after tuning.

The controller parameters were tuned sequentially considering one controller at a time. Firstly, the innermost current controller was designed for the electrical time constant of the MR damper, so that a maximum current of 1A could be attained with a desired speed and accuracy. The gain parameters were tuned separately, and the speed was optimized after attaining a fair accuracy. It was achieved by the Ziegler–Nicholas method of tuning the PI controller. After tuning of the PI controller, i.e., the current controller, the prosthesis was fitted on the subject for familiarization. When the subject was able to walk properly with certain level of comfort, the prosthesis was then considered ready to be tuned for the secondary level of control. As stated in Section 2.2, a gait cycle was segmented into 5 states (shown in Figure 7), and for each state one current reference was to be set, thereby a total of 5 parameters of the finite state controller needed to be tuned. Figure 7e,f depict the same damping as shown in Figure 8, however, for the sake of visualization, they are shown separately. It was usually straight-forward to make an initial guess of only 5 parameters. However, to avoid unnecessary discomfort to the subject, the parameters were tuned sequentially in each state depending upon the feedback from the subject and the prosthetist.

After obtaining an initial set of parameters, all the values were iteratively fine-tuned to attain a gait characteristic closer to that of a healthy individual. The knee angle was used as an essential quantitative measure to observe the performance. Subject feedback along with that of the trained prosthetist was considered primary qualitative criteria to tune the parameters. This type of tuning avoids unnecessary discomfort to the subject. The final set of current references are depicted in Figure 8.

Once the prosthesis was tuned for level walking, the subject was given sufficient training time to get proper acclimatization to it. A number of training sessions were conducted for the subject in accordance with the training methods reported in several studies [38,39,40,41]. The training was usually specific to each subject, thus the sessions were planned by a trained prosthetist keeping in view that the subject had been using a passive prosthesis for a long duration. When the subject actively started to walk on level ground, the experimental data was collected for analysis.

### 3.2. Experimental Setup

The subject was asked to walk on a 24.38 m (80 feet)-long corridor at a uniform subject comfortable speed. The initial walk of 3.05 m (10 feet) and 3.05 m of walk towards the end were excluded to avoid any deviation in biomechanical data that may arise due to acceleration and deceleration during beginning and ending, respectively. Hence the walking variables were measured over an active length of corridor of 18.29 m (60 feet) which was assumed to be uniform. 

**Figure 9:**
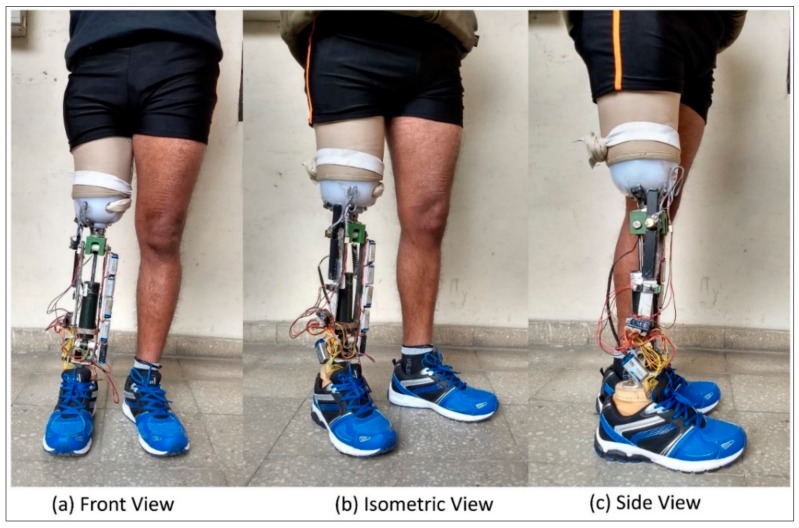
Photograph of experimental setup and subject during testing of prosthesis.

For the subject (fitted with the prosthesis as shown in Figure 9), a total of 10 trials were conducted; one trial included initial standing of 10 s and then level walking of 24.38 m (80 feet) followed by standing for 10 s. The starting, stopping, and other necessary instructions were provided verbally to the subject. After each trial, the subject was asked to take a minimum of 2 min rest which could be increased based on subject’s requirement. In order to evaluate the performance of the developed prosthesis, the data obtained from the knee goniometer, foot plantar insole, on-board clock, and the voltage and current sensors were transmitted wirelessly with a Bluetooth module to a remote desktop.

A freeware serial port terminal application (CoolTerm, version 1.4.7) was used to record the data with a sampling frequency of 100 Hz. The data was filtered in MATLAB^®^ R2015b using a low pass Butterworth filter of 4th order with a cut-off frequency of 10 Hz. The cut-off frequency of 10 Hz was preferred to filter the data as the frequency of human motion was typically less than 5 Hz. The signal was further smoothened using a 5-point moving average filter. 

## 4. Results

### 4.1. Gait Kinematics

**Figure 10:**
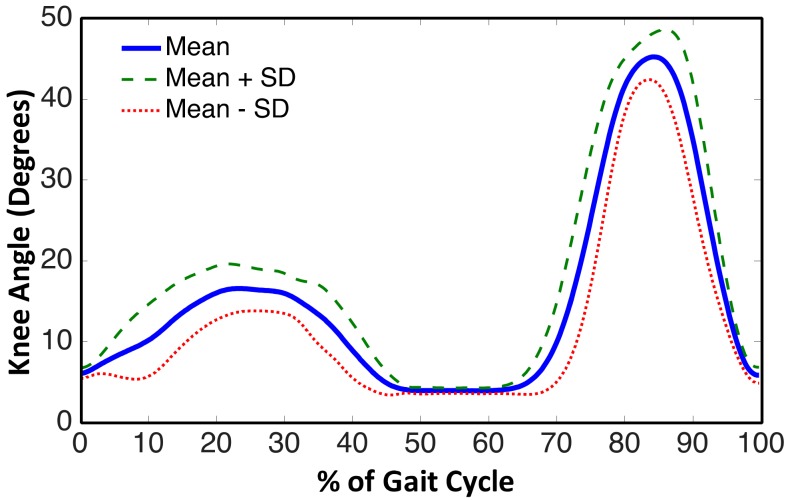
Gait kinematics measured during experiment.

Knee joint angle is the most established parameter to evaluate the kinematic performance of a prosthesis [37]. The measured knee angle from the on-board goniometer during level walk is shown in Figure 10. The prosthesis was able to demonstrate an early stance phase knee flexion-extension; at a self-selected walking speed of a gait cycle, the knee joint in early stance flexed up to 15 ± 5 degrees. The swing phase flexion was constrained up to 45 ± 7 degrees, which is below the biologically acceptable limit of 70 degrees. Combining both, the prosthesis realizes a knee angle trajectory that is closer to that of able-bodied or normal gait kinematics.

### 4.2. Power Consumption

**Figure 11:**
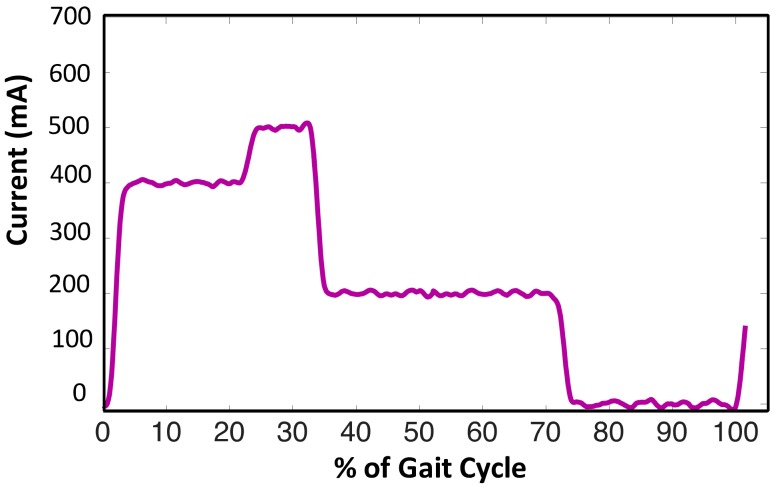
Actual current flowing in the MR damper during a typical gait cycle.

External power requirement is the major concern for both active or semi-active prostheses. To evaluate the performance of the prosthesis, the assessment of power consumption was carried out by measuring the average current through the battery. In this study, the average electrical power provided by battery bank and the consumption by MR dampers were measured by Hall Effect current sensors (ACS712). The input electrical power consumed in a gait cycle can be evaluated from Figure 11 by considering supply voltage of 9 V. In-line with the expectation, the peak power was perceived during mid-stance as the prosthesis supports entire body weight at this time. The power consumption during loading response was less than the peak power due to double support. In terminal stance, the body transfers the load to the sound limb. Hence, a moderate power was consumed during this phase. During the swing and pre-swing phases, the prosthesis consumes very little or almost no power due to very little load of prosthesis itself. An average power of 2.25 W was consumed by the MR damper during a typical gait cycle of over-ground level walking.

## 5. Discussion

### 5.1. Gait Biomechanics

The response of developed prosthesis is seen in Figure 10. It can be noticed that the gait kinematics are more similar to that of a healthy person performing a level walk. On comparison of measured knee angle to a typical gait data [29] (as shown in Figure 3), the response is quite similar to that of normal gait. Contrary to its passive counterparts, it enables the amputee to achieve knee flexion-extension during heel strike and mid stance with the required degree of stability. Such an extent of stance flexion and extension is absent in the typical gait cycle of an amputee using any passive prosthesis [38,39,40]. Controlled swing phase knee flexion-extension is also achieved; thus meeting the two necessary requirements of a prosthesis to provide able-bodied gait kinematics [35]. Though the kinematic data of healthy individuals and amputees can be compared, one should avoid trying to find a precise match between the two. The healthy gait data serves as a reference for essential characteristics only. The results presented in this study can best compared with that in the study conducted by Herr and Wilkenfeld on four unilateral transfemoral amputees using MR damper knee joints [23]. The knee joint angle of the proposed prosthesis exhibits all the characteristics similar to that of the knee angle trajectory obtained in [23]. 

The lack of smooth stance-to-swing transition is a major problem witnessed in many of the previous prostheses [11,37]. This problem is usually associated with hyper-stabilizing stance phase knee joints, such as polycentric knee joints, causing delayed initiation of swing phase. The developed prosthesis, due to its single axis design and control strategy, effectively overcomes the stance to swing transition problems. The design and control of the prosthesis also eliminates the necessity of full knee extension at the end of the swing phase as observed by Arelekatti et al. [16].

### 5.2. Control with Plantar Insole

The control of a semi-active or active prosthesis is usually achieved by highly sophisticated methods including complex sensing, data driven advanced AI, and state of the art embedded systems [23,26]. All these methods require computationally efficient software and hardware platforms associated with delicate peripherals and sophisticated electronic circuits, thereby making it a costly affair for low-income economies despite its huge potential to realize normal gait kinematics. The novel control method introduced in this study provides a low cost, effective, and efficient alternative to existing prostheses. The simple form of digital information requires almost no processing and does away with the unnecessary delay caused by Analog to Digital Convertors. Hence, it enables the designer to select a simple, low cost and low power microcontroller, a major component contributing to higher cost.

The study establishes that a foot plantar insole possesses significant information required to control the prosthesis. The basic principle of extraction of information from a plantar insole lies in ROS created by the wheel-like mechanism of the foot during locomotion, as also reported earlier [30,31]. Different ROSs are caused by different kinematic patterns. However, the kinematic data is also subject dependent but has well defined characteristic trajectory. The patterns usually vary with age, type of locomotion, and kind of locomotion impairment. This gives the opportunity to extend to other fields, such as orthoses for lower limbs. The electronically controlled prostheses are either joint angle controlled or torque controlled. The angle control approach is inherently not suitable for prostheses due to biomechanical reasons, as reported by Sup et al. [37]. On the other hand, the torque control or tracking with state impedance control schemes, have already established its suitability in active prostheses [42], but its application in semi-active prostheses [43] may not be cautious enough as semi-active prostheses reported in [24,25,26] using an MR damper do not contribute to net propulsive power during walking. Its function still remains as a passive device with impedance or damping modulation despite torque control. Hence, torque as a kinetic variable representing work and energy interaction is not suitable and is an over-applied idea for semi-active prostheses. Damping modulation can be achieved by simpler yet efficient control methods based on variables representing sufficient information of a gait cycle.

### 5.3. Cost and Weight

The developed prosthesis was designed in-house, fabricated, and assembled in the lab in India. Other than the MR damper, all the components were customized and locally manufactured with locally available materials. In an initial attempt to test the feasibility of the control scheme in less time, the fabrication of the MR damper was avoided and the most widely used MR damper (Lord Corporation Model RD-8041) was imported. The total cost of developed prosthesis is 17,000 INR excluding the damper; a suitably customized, locally designed, and manufactured MR damper is estimated to incur 5000 INR more, amounting the total cost to 22,000 INR. This cost is comparable to commonly available low cost passive prostheses which typically cost ranging from 6000 to 150,000 INR [13]. The total weight of developed prosthesis is 1.77 kilograms (kg) excluding damper and is expected to add 0.5 kg after attachment of customized locally manufactured damper. A total weight of 2.27 kg is quite lower than that of reported active and semi-active prostheses [12,23]. The component-wise manufacturing/production cost and weight is summarized in Table 4.

### 5.4. Battery Life and Power Consumption 

While importing external power to the prosthesis, the power consumption assessment is vital to its commercial acceptance. The average power consumption during a gait cycle was measured to be less than 2.5 W during over-ground level walking, whereas that of a typical active prostheses is in the range of 60–100 W for the same [37,42]. Hence, (i) the power consumption of the developed prosthesis is almost 30 times less than its active commercial counterparts, and (ii) a reduction in power requirement can enable further reduction in weight and cost. For a battery bank of 9 V and 2.4 Ahr, the battery can deliver up to 8 h of continuous supply to the developed prosthesis on a level walk. With this power arrangement, considering stride time of 2 s and stride length of 70 cm, the amputee will still be able walk 14,400 steps or a distance of around 10 km. As suggested by references, an adult walks 4000 to 18,000 steps per day [44,45]. Hence, the prosthesis is capable of operating an entire day with one time charging in a day. This is a significant advantage over active prostheses that requires ~115 Wh (Equivalent to a 9 V, 12 Ahr battery) to work all day [37]. In low-income/developing countries, where access to usual electric charging may not be available at all times, a customized provision of charging with renewable sources such as PV panel, wind etc. can be made at a minimal additional cost.

### 5.5. Prosthesis Utilization

The level of utilization is one of the parameters to evaluate the usability of any system. A system usability score (SUS) [46] has been calculated based on the questionnaire response of the subject after using the developed prosthesis. The same questionnaire has been used in the past on several rehabilitation studies [47]. The SUS score of 85 shows a promising scope for high usability of the prosthesis in Indian scenario (Table 5).

### 5.6. Implications for Commercial Applications

There are a variety of factors that affect the applicability, commercial viability, and social acceptability of a prosthesis in many low-income economies. The prosthetic needs in these countries differ based on functional demand, local availability of materials, cost, and cultural barriers [4]. As suggested, some points of consideration of appropriate technologies for prosthetics in developing countries like India primarily include low cost, local availability, consideration of local climate, suitability for local manufacturing, and durability [5,21,45,48,49]. Similar recommendations were made at the ISPO Consensus Conference on Appropriate Prosthetic Technology in developing Countries [21]. The developed prosthesis complies with most of the recommendations, and these suggested features, along with description, are tabulated in Table 6. The cost of the passive prosthesis ranges from US $125 to 1875 as per Sam et al. [9].

### 5.7. Parameter Tuning

It may appear that there are seven parameters to tune, but usually in a scenario where the prosthesis is manufactured at a facility and the patient is trained by prosthetist, the current controller parameters can be tuned by the manufacturers and the remaining five can be tuned by the prosthetist. It is justified as the prosthetist is trained and experienced at analyzing gait patterns, and therefore it is easier for he/she to judge the suitable value of the parameters. The highlight of the proposed design is the ease of fabrication in low-resource settings, and thereby can help amputees in locomotion.

### 5.8. Limitations and Future Work 

Though the prosthesis achieves its objective in terms of kinematic performance and other parameters explained earlier, there is ample scope to take it to the next level, as this work is a pilot study. Factors such as heat dissipation, the reliability of the insole sensors, and security of control to prevent falls need further attention. The developed prosthesis design is in its initial stages and further modifications need to be made to bring the design out of the lab setting. A locally manufactured MR damper needs to be fixed to the prosthesis in order to extensively evaluate the performance of the developed prosthesis on a large number of the targeted population as the prosthesis has been tested on single amputee only. A more aesthetic make over with suitable casing and fitting could unveil its humanoid appearance. The present design and control method addresses level walking with a single speed only, a variable speed adaptation would enhance its maneuverability and user friendliness. The future work would include the implementation of the above left-over points with design, development of control scheme for variable speed adaptation, and thorough testing of the modified design on more subjects.

## 6. Conclusions

A novel low-cost lower limb prosthesis based on sensors placed in a plantar insole was presented in this work. The hardware with suitable control architecture enabled the trans-femoral amputee to attain able-bodied gait kinematics. The results are encouraging and provide a new dimension to the control philosophy of proposed prostheses. The study presents the initial prototype and hence may need thorough testing and related modifications before commercialization. The developed prosthesis is an optimum choice for lower limb amputees in low-income or low-resource settings as well as in developing countries. The attractive features of low cost, durability, and able-bodied gait performance are expected to expand its commercial reach beyond India to other low and medium income developing countries.

## Figures and Tables

**Table 1:** Sensor states during different phases of gait.

Gait Segment	S1	S2	S3	S4
Loading Response	1	0	0	0
Mid-Stance	1	1	0	0
Terminal-Stance	0	1	0	0
	0	1	1	0
	0	1	1	1
Pre-Swing	0	0	1	1
	0	0	0	1
Swing	0	0	0	0

**Table 2:** Details of the embedded system components.

Components	Rating
MOSFET IRF 540N	100 V, 33 A
Diode SR 360	60 V, 3 A
Hall current sensor ACS 712	230 V, 5 A
Opto-coupler MCT2E 4050	3 V, 50 mA
MR damper RD 8041	12 V, 1 A
Filter capacitor	50 V, 100 µF
Filter inductor	1 A, 33 µH

**Table 3:** Details of the amputation and prosthesis.

**Stump Length**	22 cm
**knee axis w.r.t.**	34 cm
**Ischial bone**	
**Knee axis to floor**	47 cm
**Socket design**	Ischial containment socket
**Prosthetic foot**	SACH foot

**Table 4:** Manufacturing cost and weight of various components.

Component	Production Cost (INR)	Weight (kg)
Knee joint & mechanical structure ^a^	7000	0.810
Sensorized plantar insole	1000	0.015
Sach Foot	900	0.710
Embedded electronics	2000	0.085
Rechargeable batteries	5000	0.150
Total	17,000	1.770

^a^ Excluding MR damper.

**Table 5:** System usability scale (SUS) score ^a^.

Questionnaire Item	Weight
I think that I would like to use this prosthesis frequently.	5
I found the system unnecessarily complex.	1
I thought the system was easy to use.	5
I think that I would need the support of a technical person to be able to use this system.	1
I found the various functions in this system were well integrated.	4
I thought there was too much inconsistency in this system.	2
I would imagine that most people would learn to use this system very quickly.	3
I found the system very cumbersome to use.	2
I felt very confident using the system.	5
I needed to learn a lot of things before I could get going with this system.	2
SUS Score	85

^a^ The SUS scores range from 1 (“strongly disagree”) to 5 (“strongly agree”). The SUS score was calculated based on the responses following Brooke [46].

**Table 6:** Technical features and descriptions based on recommendations of ISPO consensus conference on appropriate prosthetic technology in developing countries [5,20,21].

Technical Feature	Description
Low cost	An expected cost of 22,000 INR is very low keeping in view the performance and features offered. The mass production of prosthesis would further cut the cost. Those already using modern passive prostheses can afford such a cost and others can be supported by governmental schemes as well as non-profit organizations already working in this field.
Technical functionality	It serves the basic functions of providing stability during stance phase and controlled flexion during the swing phase of a gait cycle. It successfully prevents knee buckling on heel strike and delayed transition from stance to swing phase.
Biomechanical appropriateness	The feasibility of early stance flexion-extension of knee joint makes it biomechanically appropriate; incorporation of suitably damped swing phase can enable the amputee to match its speed with that of the sound limb.
Light weight	An expected total weight of 2.270 kg of prosthetic leg is comparable to commercially available modern passive prostheses in India. The usual weight of a passive prosthetic leg varies between 2 to 3 kg in India due to physiological parameters of Indian population.
Use of locally available materials	The prosthesis is made up of SS and Metalon which are widely available in most parts of India. Both the materials are affordable, high strength, and durable for a long period.
Consideration for local climate	The prosthesis is designed keeping in view the hot and humid conditions of the Indian subcontinent. The material used in the prosthesis can bear several times the excessive weather conditions including water, heat, and dirt.
Durable	The simple design and suitable material selection can make a prosthesis durable in developing countries. ISO-10328 specifies that a prosthetic leg/knee must endure 3 million cycles which is equivalent to approximately 3 years of use.
Simple to process and repair	The design of the prosthesis is modular and each part can be replaced or repaired separately. The components are reproducible by local personnel and can be manually fabricated. The prosthesis is simple to process and repair using local production capability.
